# Delayed hemothorax following blunt thoracic trauma: a case report

**DOI:** 10.1186/s13019-024-02914-5

**Published:** 2024-06-27

**Authors:** Astrid Carolina Álvarez-Ortega, Alcibíades Aranda-Hoyos, Jose Alejandro Posso-Nuñez, Carlos Alejandro García-González, Juan Carlos Puyana, Álvaro Ignacio Sánchez-Ortiz, Mauricio Velásquez-Galvis

**Affiliations:** 1 Clinical Research Center, Fundación Valle del Lili, Kra 98 No. 18 - 49, Cali, 760032 Colombia; 2 School of Medicine, General Surgery, Universidad del Cauca, Popayán, 190003 Colombia; 3 Department of Radiology and Diagnostic Imaging, Fundación Valle del Lili, Kra 98 No. 18 - 49, Cali, 760032 Colombia; 4 Director for Global Health-Surgery, University of Pittsburgh, UPMC Presbyterian, F1263 200 Lothrop Street Pittsburgh, Pittsburgh, PA 15213 U.S.A.; 5 Department General Surgery, Division of General Thoracic Surgery, Fundación Valle del Lili, Kra 98 No. 18 - 49, Cali, 760032 Colombia

**Keywords:** Hemothorax, Sepsis, Rib fractures, Thoracic surgical procedures

## Abstract

**Background:**

Late hemothorax is a rare complication of blunt chest trauma. The longest reported time interval between the traumatic event and the development of hemothorax is 44 days.

**Case presentation:**

An elderly patient with right-sided rib fractures from chest trauma, managed initially with closed thoracostomy, presented with a delayed hemothorax that occurred 60 days after initial management, necessitating conservative and then surgical intervention due to the patient’s frail condition and associated complications.

**Conclusions:**

This case emphasizes the clinical challenge and significance of delayed hemothorax in chest trauma, highlighting the need for vigilance and potential surgical correction in complex presentations, especially in the elderly.

**Supplementary Information:**

The online version contains supplementary material available at 10.1186/s13019-024-02914-5.

## Background

Blunt chest trauma can lead to mortality rates of up to 25% in older patients [1], even following low-energy mechanisms [2]. Late hemothorax, a rare complication, exhibits a prevalence up to 12.1% [3]. Two definitions of late hemothorax exist. Shorr et al. [4] characterized it as a hemothorax that manifests 24 h after the traumatic event without being discernible in initial examinations. Ritter et al. redefined it as hemothorax diagnosed in subsequent studies within two hours from the initial examination [5]. The longest reported time interval between the traumatic event and the development of hemothorax is 44 days [5]. In most cases, surgical management is not required. We report a case of bleeding at 60 days requiring surgical management.

## Case presentation

A 79-year-old male was admitted to the emergency department with persistent pain on the posterior right chest wall after a fall from a stepladder while changing a lightbulb that had occurred 5 days before presentation. Past medical history included heart failure, hypertension, type 2 diabetes mellitus, aortoiliac aneurysm correction, and biological mitral valve replacement 10 years ago. Before admission, his medications included nifedipine, clonidine, timolol, and metformin.

Upon arrival, his temperature was 36 °C, pulse 75/min, breaths 16 /min, blood pressure 102/63 mmHg and oxygen saturation 94%. Physical examination showed bruising along the right costal margin. Pulmonary sounds were absent in the right hemithorax. Computed tomography (CT) scan reported non-displaced transverse fractures in the middle third of the right seventh, eighth, ninth, and 12th costal arches, and depressed rib fractures of the 10th and 11th arches (Image 1 A), along with a massive hemothorax. Treatment included closed thoracostomy, with drainage of 1200 cc upon insertion of the chest tube, analgesia, and respiratory therapy. On the fifth day of inpatient management, with symptom improvement, stable hemoglobin (Hb) levels (Table 1), and a chest X-ray showing minimal residual hemothorax (Image 2), the tube was removed, and the patient was discharged.

**Fig. 1:**
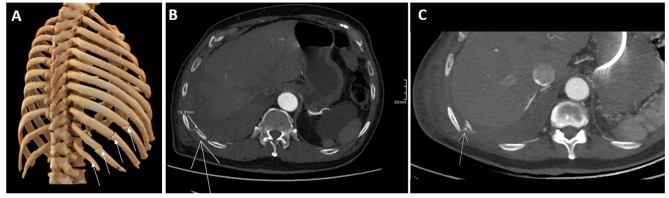
Temporal Evolution of Post-Traumatic Chest Injuries. A: 3D CT scan reported non-depressed rib fractures of the right 7th, 8th, 9th, and 12th costal arches and depressed rib fractures of the 10th and 11th arches (arrows). B: 11 days after the trauma, an irregular ectasia (arrow) of 2 cm of the 10th right intercostal artery is seen. C: A 3 mm nodular image is seen in the arterial phase (arrow) adjacent to the right rib fracture site, suggesting a pseudoaneurysm within the 10th right intercostal artery

**Fig. 2:**
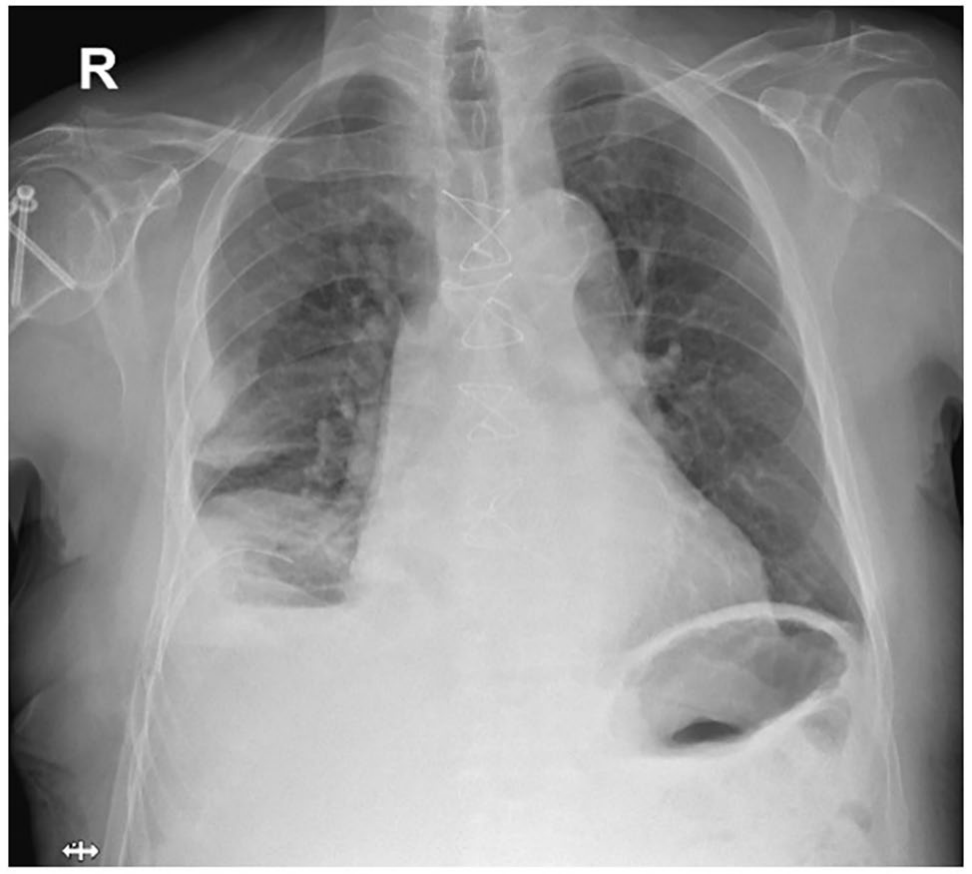
Chest X-Ray at 10th post-trauma day: lung expansion with right sided pleural thickening, a right basal opacity that may correspond to a residual pleural effusion.

**Table 1:** Daily record of laboratory parameters and drainage quantification in key moments of the patient’s course of disease

	Post-trauma day														
Laboratory Results	Day 5	Day 8	Day 11	Day 15	Day 23	Day 28	Day 29*	Day 33	Day 58	Day 60*	Day 65	Day 68	Day 76	Day 91	Day 101
Hemoglobin (g/dL)	12.1	9.6	9.4	5.7	7.5	6.3	7.4	8.7	6.2	8.5	6.7	7.3	8.5	8.3	5.5
Platelets (per µL)	141,000	128,000	202,000	179,000	155,000	150,000	86,000	36,000	118,000	105,000	90,000	95,000	74,000	244,000	119,000
Leukocytes (per µL)	7570	13,690	17,500	20,310	19,420	14,570	15,550	9690	7360	7710	5380	9080	13,870	7710	9760
C Reactive Protein (mg/dL)	N/A	N/A	N/A	15.72	9.24	13.15	N/A	20.92	5.05	N/A	N/A	9.41	10.69	N/A	N/A

*Laboratory results displayed on the days of first and second VATS correspond to presurgical values

**Table 2:** Timeline of key events

Post-trauma day	Events	Details
5	First admission	
10	Patient discharged	
11	Readmission	First CTA of the readmission period, piperacillin-tazobactam initiation
15	Transfer to the ICU	Antibiotic therapy adjustment: IV meropenem + IV vancomycin
23	GI bleeding and Upper GI endoscopy	Fundal ulcer, Forrest IIa classification, 2 endoclips for hemostasis
28	Shock	Double vasoactive requirement to stabilize
29	First VATS	
33	Nosocomial pneumonia	Episode of desaturation, increased secretions lead to fiber optic bronchoscopy to clear the airways and a sample is taken, growing KPC
58	Insertion of permanent dialysis catheter + possible 10th right intercostal artery bleeding	Second CTA of the readmission period
60	Second VATS + sacral ulcer debridement	Active bleeding from the 9th right intercostal artery found; 2.5 L hemothorax drained
65	VATS-lung decortication sample positive for B. cepacia	TMP-SMX therapy initiation
68	Shock	Requires invasive mechanical ventilation
76	C. difficile testing positive	Oral vancomycin therapy started + IV metronidazole is started on day 77
91	Delirium	
102	Vasoplegic shock and patient’s demise	

A day after discharge, the patient returned with symptoms of dyspnea and oozing bleeding from the thoracostomy site. Blood pressure measured at 123/55 mmHg, pulse rate was 101/min, respiratory rate was 22/min, oxygen saturation was 95% and the temperature was 36.5 °C. Among abnormal laboratory findings were 17,500 leukocytes, 9.4 g/L Hb, 202,000 platelets, 1.54 mg/dL serum creatinine (sCr) and 43.5 mg/dL blood urea nitrogen (BUN). A transthoracic echocardiogram showed preserved left ventricle ejection fraction (LVEF), but a dysfunctional mitral valve, to which a transesophageal echocardiogram (TEE) was ordered after an additional finding of increased NT-pro-BNP. CT angiography (CTA) demonstrated a small residual clotted hemothorax, without any sources of active bleeding (Image 1B). A superimposed bacterial infection of the residual hemothorax was suspected. Given the patient’s extensive medical history and high risk for postoperative pulmonary complications, as calculated by use of the ARISCAT Score, a conservative management approach was taken, including intravenous (IV) piperacillin-tazobactam, deep venous thrombosis (DVT) prophylaxis with subcutaneous enoxaparin and gastrointestinal (GI) bleeding prophylaxis with IV omeprazole. On post-trauma day 13, TEE was performed, finding preserved LVEF, functional mitral valve and atrial flutter, with successful return to sinus rhythm after cardioversion. Although continuous, low-flow, bleeding from the thoracostomy site prevailed; clinical condition remained stable over the next days. On day 15 post-trauma, the patient deteriorated with somnolence, hypotension and dyspnea with laboratory findings of multisystem failure. The patient was transferred to the intensive care unit (ICU), with a suspected shock of mixed infectious and hemorrhagic etiology, labs showing leukocytosis, severe anemia with Hb 5.7 g/L, grade III acute kidney injury among other findings (Table 1). Management at this point included invasive mechanical ventilation, packed red blood cell (RBC) transfusions and adjustments in antibiotic therapy with initiation of IV vancomycin and meropenem. During ICU stay, the patient had stable ventilatory parameters but with vasoactive drug requirement and anemia despite packed RBC transfusions. A new CTA was performed, as there were no other clear sources of bleeding, finding a mild increase in size of the known right loculated hemothorax but no clear signs of a source for embolization or surgical management (Image 1 C). On day 16, the patient started on dialysis, as there was no response to the use of diuretics. After an enema performed for constipation, on day 23, black, tarry stools were expelled and the patient was taken to upper GI endoscopy, where two endoclips were placed for the management of a Forrest classification IIa, gastric fundal ulcer. An improvement in the patient’s condition was seen until day 28 post-trauma, where the patient exhibited sudden hemodynamic compromise, with double vasopressor requirement and one unit of packed RBC transfused. After medical optimization, on day 29 post-trauma, the patient underwent right lung decortication by video-assisted thoracoscopic surgery (VATS), finding lung entrapment and old clots, without active bleeding.

Post-surgical care continued at the ICU. On day 33 the patient coursed with an episode of desaturation and abundant aspiration of orotracheal secretions, emergency fiber optic bronchoscopy was performed to manage secretions and obtain samples for infectious testing. Samples returned positive for carbapenemase-producing K. pneumoniae (KPC), ceftazidime-avibactam was started. Tracheostomy was performed on day 43 and initial surgical lavage and debridement for a developing sacral ulcer. The patient´s condition improved marginally, and by day 58 he had initiated physical and respiratory rehabilitation in the ICU after successfully weaning off mechanical ventilation. The same day, due to ongoing dialysis requirement, placement of a permanent catheter was performed.

On day 58 the patient suddenly presented with hypotension, a 2-point decrease in hemoglobin, and a mild lactate increase (Table 1). A new CTA ruled out bleeding from the catheter insertions but indicated a massive right hemothorax, with possible bleeding from the 10th intercostal artery adjacent to a known rib fracture (Image 3). After medical optimization, on day 60, the patient underwent a second VATS, revealing an active bleeding from the intercostal artery successfully managed through selective ligation (video 1).

**Fig. 3:**
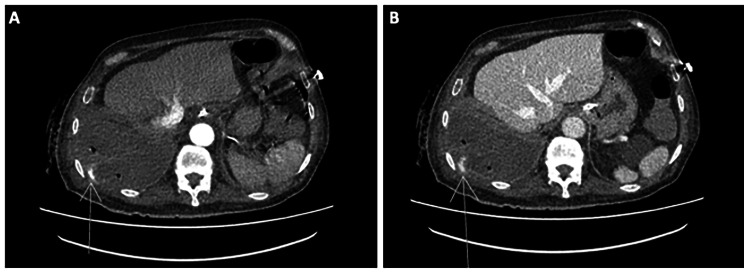
Post-Traumatic CT Angiography evolution: Day 58. In the tenth right intercostal space, contrast medium extravasation is observed in the arterial phase. **A**: within the middle third of the intercostal artery, which intensifies in the venous phase. **B**: as a sign of active bleeding.

By day 65 clinical condition had improved gradually, leading to the discontinuation of invasive mechanical ventilation and vasopressor support, allowing physical and pulmonary rehabilitation. In the same day, culture of surgical samples from second VATS returned positive for trimethoprim-sulfamethoxazole sensitive B.*cepacia*, and therapy was adjusted accordingly.

The prolonged hospital stays, and physical decline led to the development of a sacral ulcer, necessitating debridement and cleansing on multiple occasions. On day 76, the patient manifested abdominal pain and distension, hypotension, altered mental status, and prolonged capillary refill time. Thoracostomy drainage was negligible, and no apparent sources of GI bleeding were found. Fecal sampling returned positive for C. difficile, IV metronidazole and oral vancomycin were started.

Despite clinical efforts, the patient continued to have anemia with multiple transfusion requirements, low tolerance for pulmonary and physical rehabilitation, anuric kidney failure and on post-trauma day 102, refractory septic shock ensued with subsequent asystole and the patient’s demise after withholding resuscitation maneuvers.

## Discussion and Conclusions

This case involves an elderly patient with multiple comorbidities who sustained blunt thoracic trauma, resulting in multiple rib fractures (MRF) and hemothorax without clear indications for surgical management. Initially, the patient responded to conservative management of the MRF and tube thoracostomy for the hemothorax. However, the clinical course became erratic, characterized by anemia, acute renal injury, GI bleeding and multiple infectious complications. Notwithstanding, active bleeding from an intercostal artery was not detected until post-trauma day 58, which delayed surgical management.

The management of rib fractures and mild to moderate hemothorax are controversial. This patient was not considered for surgical stabilization of the rib fractures because the fracture patterns (non-displaced or minimally displaced) combined with the patient´s comorbidities, suggest a greater risk than the benefit in undergoing surgical stabilization [6]. Traditionally, the management of hemothorax includes chest tube placement. Observational data has suggested that expectant management of hemothorax following blunt chest trauma may be an appropriate strategy in stable patients [7].

Delayed hemothorax is a rare complication from blunt chest trauma, predominantly affecting the elderly. About 12.3% of cases result in delayed hemothorax, usually within 14 days from the day of trauma [1]. MRF are associated in 92% of cases. Recent reports detail cases from 30 up to 44 days post-trauma [3]. Delayed hemothorax represents significant morbidity and functional decline in this group of patients. This patient has predictors for delayed hemothorax. A propensity cohort study proposed a clinical prediction score for delayed hemothorax. According to its findings, both an age > 70 years and the presence of three or more rib fractures are factors present in the patient that may associate to the occurrence of this complication [1]. However, age, frailty and multiple comorbidities configured a higher risk of postoperative complications, with some presenting in this case, including acute renal injury and multiple infectious hits.

Hemothorax is common in patients with rib fractures [8]. The utility of routine evacuation of hemothorax following blunt trauma in general remains controversial. Blunt etiology of hemothorax is considered less likely to result in infection; however, retained hemothorax following blunt trauma is an established risk factor for empyema [9]. Surgery is needed in 0.4% of cases, as most cases are managed through closed thoracostomy [5]. In this case, due to deteriorating hemodynamics, VATS was required on two occasions: first, to evacuate the coagulated hemothorax suspected of causing infectious complications, and a second VATS to address active bleeding in the right hemithorax. The latter revealed an actively bleeding intercostal artery on post-trauma day 60.

Our hypothesis for the explanation of the delayed presentation of hemothorax includes the clinical predictors of the patient, the movement of unfixed rib fracture fragments leading to displacement that could occur during or after respiratory therapy. The mechanism involves displaced rib fragments, causing vessel erosion [1, 3, 4]. This, coupled with the rare, retrospective finding of a pseudoaneurysm of the same intercostal artery (Image 2 C) during documental revision for this report may underlie the intrathoracic bleeding. An earlier identification of the imaging findings could have provided an opportunity for angioembolization or surgical management, which could have mitigated the patient’s overall disease burden.

The patient in this report experienced multiple infectious events, culminating in a C. difficile infection. Advanced age, elevated CRP levels, and sepsis have been identified as predictors of mortality in such cases, which were all present in this patient’s context and outcome [10]. Numerous studies highlight advanced age as a risk factor for severe C. difficile infection outcomes, with analyses showing a significant independent association between age ≥ 80 years and mortality, yielding an odds ratio of 5.5 [11].

This case highlights a delayed presentation of hemothorax, the longest reported to our knowledge. It also establishes the bleeding source and successful hemostatic control through VATS. Healthcare providers should be vigilant, especially among older adults displaying post-trauma symptoms long after blunt trauma.

### Electronic supplementary material

Below is the link to the electronic supplementary material.

**Supplementary Material 1: Video 1:** VATS 60 days post-trauma. Video assisted thoracoscopy surgery (VATS) exhibiting active bleeding from a right 10th intercostal artery.

## Data Availability

No datasets were generated or analysed during the current study.

## Abbreviations

CT: Computed tomography
Hb: Hemoglobin
sCr: Serum Creatinine
BUN: blood urea nitrogen
LVEF: Left Ventricle Ejection Fraction
TEE: Transesophageal Echocardiogram
CTA: CT angiography
DVT: Deep Venous Thrombosis
IV: Intravenous
GI: Gastrointestinal
ICU: Intensive Care Unit
RBC: Red Blood Cell
VATS: video-assisted thoracoscopic surgery
KPC: carbapenemase-producing K. pneumoniae
MRF: Multiple Rib Fractures;
